# Microstructure-Property Correlation and Its Evolution during Aging in an Al_4.4_Co_26_Cr_19_Fe_18_Ni_27_Ti_5.6_ High-Entropy Alloy

**DOI:** 10.3390/ma16072821

**Published:** 2023-04-01

**Authors:** Florian Biermair, Francisca Mendez-Martin, Vsevolod I. Razumovskiy, Franco Moitzi, Gerald Ressel

**Affiliations:** 1 Materials Center Leoben Forschung GmbH, Roseggerstraße 12, 8700 Leoben, Austriagerald.ressel@mcl.at (G.R.); 2 Department of Materials Science, Montanuniversität Leoben, Franz-Josef Straße 18, 8700 Leoben, Austria

**Keywords:** compositionally complex alloy, characterization, precipitation strengthening, solid solution strengthening, yield strength prediction

## Abstract

The efficient energy use in multiple sectors of modern industry is partly based on the efficient use of high-strength, high-performance alloys that retain remarkable mechanical properties at elevated and high temperatures. High-entropy alloys (HEAs) represent the most recent class of these materials with a high potential for high-temperature high-strength applications. Aside from their chemical composition and microstructure-property relationship, limited information on the effect of heat treatment as a decisive factor for alloy design is available in the literature. This work intends to contribute to this research topic by investigating the effect of heat treatment on the microstructure and mechanical performance of an Al_4.4_Co_26_Cr_19_Fe_18_Ni_27_Ti_5.6_ HEA. The solution annealed state is compared to aged states obtained at different heat treatment times at 750 °C. The temporal evolution of the matrix and the γ’-precipitates are analyzed in terms of chemical composition, crystallography, size, shape, and volume fraction by means of scanning electron microscopy, transmission electron microscopy, and atom probe tomography. The yield strength evolution and strength contributions are calculated by classical state-of-the-art models as well as by ab-initio-based calculations of the critical resolved shear stress. The findings indicate promising mechanical properties of the investigated alloy and provide insight not only into possible strengthening mechanisms but also into the evolution of main phases during the heat treatment.

## 1. Introduction

High-entropy alloys (HEA) have been a subject of an intense research effort over nearly the past two decades. These alloys exhibit unique combinations of mechanical, corrosion, thermal, and other properties, which make them attractive candidates for various applications. A special subcategory of HEAs has been given special attention in the past decade. These are the so-called compositionally complex alloys (CCAs) [1,2,3,4]. Unlike traditional HEAs, CCAs have substantial deviations from the equiatomic composition but still have more than one principal element to differentiate them from classical alloys. Similarly to HEAs, CCAs normally consist of five or more alloying elements with concentrations of at least five at.% and usually have a configurational entropy of 1.5 R (R is the ideal gas constant) or above. The high configurational entropy from mixing major elements aims to stabilize phases which would be beneficial for selected alloy properties at elevated and high temperatures [2,5]. However, according to George et al. [6], the entropy cannot be ascribed to be the main factor defining the structure and properties of these alloys. Since the stabilizing effect of configurational entropy cannot fully compensate for the driving forces for secondary phases, they cannot be suppressed in most cases [7,8]. Generally, precipitates can have a significant contribution to the material strength [9,10], which is being exploited in the design of modern CCAs [4,11,12,13,14]. This is often opposed to the classical single-phase HEAs.

CCAs are considered a promising material class for construction applications, requiring components that are exposed to high temperatures and mechanical loads, where they are envisioned to be a possible replacement for classical wrought Ni-based superalloys [15]. A good example is the Al_3.31_Co_27.27_Cr_18.18_Fe_18.18_Ni_27.27_Ti_5.78_ alloy [16], comprising a γ/γ’ microstructure similar to classical Ni-based superalloys consisting of a disordered solid solution FCC matrix (γ-phase) and coherent, ordered L1_2_ precipitates (γ’-phase). This alloy has already been partly investigated in the peak aging condition [17,18], including aging at various temperatures [19]. However, the composition of the γ/γ’ microstructure may undergo significant changes during heat treatment, calling for the necessity of detailed investigations regarding the behavior of such alloys during tempering. In particular, the detailed knowledge of the evolution of precipitate and matrix properties, including size, chemistry, phase stability, and calculated mechanical properties of the matrix phase at different states during aging, is crucial for a better understanding of the microstructure-property relationship.

In the present work, the promising precipitation strengthened CCA Al_4.4_Co_26_Cr_19_Fe_18_Ni_27_Ti_5.6_, exhibiting increased Al content compared to the above-described Al_3.31_Co_27.27_Cr_18.18_Fe_18.18_Ni_27.27_Ti_5.78_ alloy, is characterized for the first time in detail after different aging durations by a combination of state-of-the-art methods. These methods allow for a detailed evaluation of alloy constitution and phase identification as well as detailed chemical evolution of present phases and their comparison to classical Ni-based superalloys in order to pronounce structural differences. APT-informed ab initio calculations, in combination with state-of-the-art models, finally allow conclusions to be drawn about the microstructure-property correlations of this novel alloy. 

## 2. Methods

### 2.1. Experimental Details

In the present work, a CCA was investigated and compared to the results of the state-of-the-art Ni-based superalloys, IN718 and IN939, from the literature [20,21]. Their overall chemical compositions (in at.-%) are given in Table 1.

The CCA (Al_4.4_Co_26_Cr_19_Fe_18_Ni_27_Ti_5.6_ alloy) was melted under a vacuum and subsequently cast into a cast iron mold in order to produce an alloy with a total mass of approximately 40 kg. A more detailed description of the production is given in [22]. The ingot was machined into disks of approximately 1 cm in height and homogenized at 1150 °C for 12 h in air. Specimens were cut from the homogenized disc, solution annealed, and subsequently, water-quenched (WQ) and aged for 10, 30, and 50 h at 750 °C. Specimens in WQ- and aged states were then sliced into smaller cuboids. It should be noted that the varying heat treatments of the alloys from the literature compared to the CCA in Section 3.1.2 and Section 3.1.3 can be found elsewhere [20,21,23,24,25]. For the chemical composition comparison in this sections, IN718 was heat treated at 718 °C for 8 h [20], whereas IN939 was heat treated at 1000 °C for 6 h and 800 °C for 16 h [21]. Furthermore, for the particle size and volume fraction comparison in Section 3.1.2, IN718 was heat treated at 720 °C for 6 h and additionally at 620 °C for 6 h [23], whereas IN939 was heat treated at 1000 °C for 6 h and additionally at 800 °C for 16 h for comparison of the size [24] or at 850 °C for 6 h for the volume fraction [25].

For scanning electron microscopy (SEM) analysis, the surface was prepared by ionslicing with a Hitachi IM4000+ and plasma cleaned with an Evactron^®^ E50 SoftCleaner^®^ (XEI Scientific Inc., Redwood City, CA, USA). SEM characterization of the cross-section was performed on a GeminiSEM^®^ 450 (Carl Zeiss SMT, Oberkochen, Germany). The Olympus Stream Motion software was used to separate the matrix from precipitates on two representative SEM images from the intragranular region for each state. With this separation, the size and volume fraction (a ratio of areas of precipitates to the whole corresponding sample area) were determined by optical methods.

For transmission electron microscopy (TEM) characterization, a specimen with a thickness of 0.1 mm was produced via cutting and grinding, which subsequently was dimpled and Ar+ ion polished by using a Gatan PIPS II precision ion polishing system to achieve an electron transparent material. For the TEM investigation and selected area diffraction (SAD), a JEOL 2200 FS with an acceleration voltage of 200 kV was used. 

For the atom probe tomography (APT), rod-like specimens were cut and prepared by electropolishing to get a final tip geometry of 10–20° shank angle and less than 100 nm of diameter. The APT measurements were performed on one tip for each state with a LEAP 3000 X HR System from Cameca at 60 K and with 20%PF, 200 KHz, and 0.5% target evaporation. For tip reconstruction, the software IVAS 3.8 from Cameca was used. For the distinction between matrix and precipitates, isoconcentration surfaces of 42.5 at.% Ni were used to evaluate the precipitate size and volume fractions. To this end, in each tip, the ratio of volumes that were separated by 42.5 at.% Ni isoconcentration surfaces to the whole tip volume were determined as the volume fraction of precipitates. It should be noted that, for the chemical composition of the matrix, a 26 at.% Ni isoconcentration surface was used for evaluation, whereas for the chemical composition of the precipitates, an isoconcentration of 50 at.% (45 at.% for WQ state) was chosen. The obtained chemical compositions of the matrix, as well as the particle’s sizes from APT, were used for the computational calculations in Section 3.2.1 and Section 3.2.2. 

Compression tests of the CCA on cylindrical specimens with dimensions of 8 mm diameter and 12 mm height of the 50 h aged state were performed on an Instron 8803 with a strain rate of 2.5 × 10^−4^ s^−1^. Specimens were heated to the testing temperature inductively, and the tests were performed to a maximum strain of 2%, where the strain was measured by a laser extensometer.

### 2.2. Computational Details

Electronic structure calculations were performed using the exact muffin-tin orbital (EMTO) [26] code (Lyngby version [27]) implementing a Green’s function-based DFT methodology combined with Coherent Potential Approximation (CPA) and the full charge density (FCD) formalism [28]. The paramagnetic state of metallic alloys was described using the disordered local moment (DLM) approach [29].

Finite-temperature electronic, magnetic, and phononic effects were considered. One-electron entropy was calculated using energy contour integration weighted by the Fermi function. The finite-temperature longitudinal spin fluctuations (LSF) were described with a semiclassical model [30], which amounts from adding an entropic contribution $-TS_{mag}\left[M\right]$ to the electronic free energy for the Ni, Cr, Fe, and Co components, which exhibit itinerant magnetism. The magnetic entropic contributions were chosen for each component based on fixed spin-moment calculations. Only for Ni, Co, and Cr, $S_{mag}=3\log M$ and for Fe, $S_{mag}=\log\left(M+1\right)$ was added.

The phonon free energy was calculated within the Debye-Grüneisen model, with the parameters derived from the equation of state calculations at the respective temperatures. We employed a semiempirical correction to the exchange-correlation by adding a concentration-weighted pressure to the equation of state [31,32].

The critical resolved shear stress contribution (CRSS) ${\Delta \tau }_{SSS}$ is due to a solute-dislocation interaction, determining solid solution strengthening (SSS), and is given by the Varvenne-Curtin model [14] that was used in the same way as in [33] with materials parameters obtained from DFT calculations. The concentration derivatives of the alloy volumes were calculated by a series of CPA calculations for systems with small deviations in the concentrations from the original alloy. For each elemental component, four calculations were performed where the concentration of the respective element is changed by −δ, −δ/2, δ/2, and δ (δ = 0.0005) from the original alloy. The concentration ratios among the remaining elements were kept constant. For an N-element alloy, 4 × N + 1 calculations were performed, including the original alloy composition. The linear elastic constants were obtained from volume-conserving monoclinic and orthorhombic distortions following the computational details described in [34,35].

The formation energy was calculated as the difference of the Helmholtz free energy of the alloy, including the entropy of mixing and the total energy of its components in their respective ground states at 0K. Fcc Al and hcp Ti were considered to be non-magnetic with a = 4.0476 Å and a = 2.941 Å, c = 4.668 Å, respectively. The spin-density wave magnetic state of bcc Cr was approximated by a collinear anti-ferromagnetic state (AFM) of [001] type with a = 2.8826 Å. Hcp Co, bcc Fe, and FCC Ni were calculated as ferromagnetic with spin-polarized calculations. The respective lattice constants were a = 2.5030 Å, c = 4.0574 Å, a = 2.8670 Å, and a = 3.5159 Å, respectively. The entropy of mixing was approximated by an ideal solution: $S_{mix}=-R\sum c_{i}ln(c_{i})$, where $c_{i}$ is the atomic fraction of the i^th^ element in the alloy.

## 3. Results

### 3.1. Microstructure Characterization

#### 3.1.1. Alloy Constitution and Phase Identification

**Figure 1:**
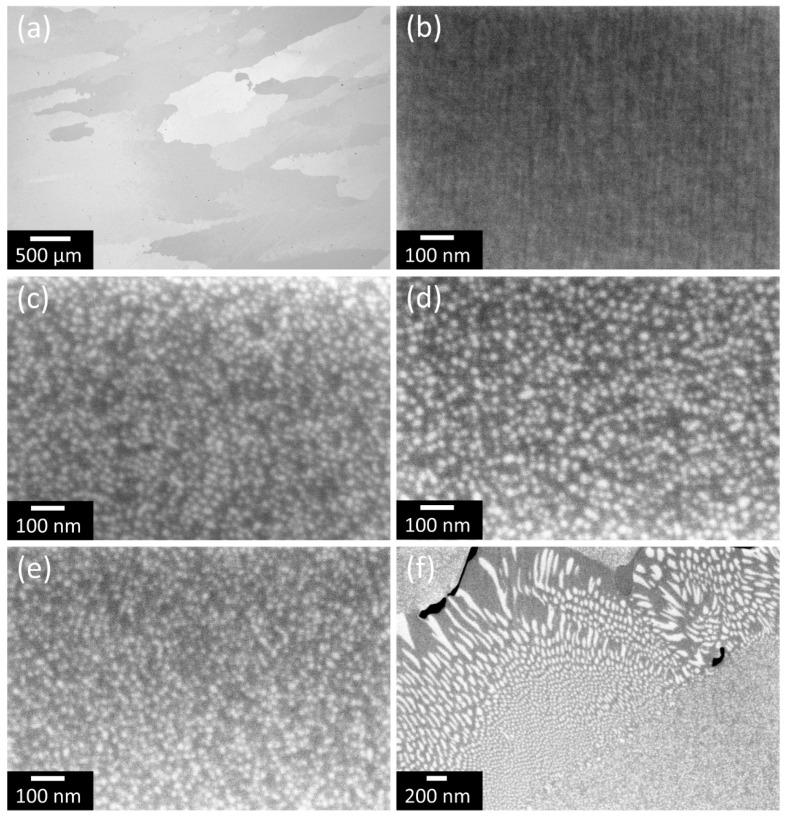
(**a**) SEM backscatter image of produced material with low magnification and SEM images of the alloy with higher magnification in the as-cast state (**b**) and after aging for 10 (**c**), 30 (**d**), and 50 h (**e**,**f**) at 750 °C. In (**a**), grains with approximate dimensions in the range of millimeters can be seen. In (**b**), no precipitates can be observed. In (**c**–**f**), precipitates of different sizes can be seen.

The alloy constitution in this work is identified by SEM and TEM. In Figure 1a, the grain size of the casted alloy is in the size of millimeters for all considered states. For characterization of the microstructure evolution on the nanometer scale, SEM images of the WQ and the aged states are shown in Figure 1b–e. Bright-appearing precipitates are observable in all aged states (Figure 1c–f), whereas a supersaturated solid solution without precipitates can be assumed in the WQ state (Figure 1b). The precipitates in sizes of tens of nanometers are homogeneously distributed in the matrix bulk of the grains, whereas the matrix is present in channels between these precipitates. Furthermore, in the proximity of grain boundaries, the precipitates and matrix channels are larger than in bulk, and the precipitates have an elongated appearance with lengths of up to 650 nm and widths of up to 100 nm (Figure 1f), which has already been discussed elsewhere [36]. The widths of the matrix channels partially exceed the largest precipitate sizes there. In Figure 1f, the black-appearing phases can also be observed. These phases are most likely nitrides or sulfides, as already observed in the homogenized state of the alloy in [22].

**Figure 2:**
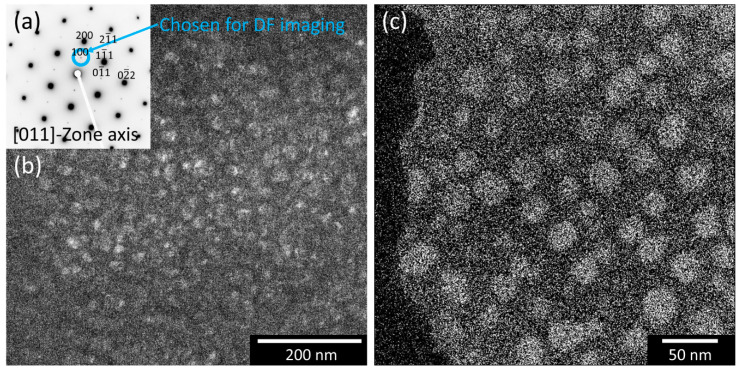
SAD (**a**), TEM DF image (**b**), and EFTEM image for Al (**c**) of the alloy after 50 h of aging. The presence of γ’ precipitates can be seen.

In order to investigate the crystallography of present phases, TEM characterization has been carried out. In Figure 2, a diffraction pattern (Figure 2a) with the corresponding TEM dark field (DF) image (Figure 2b) and an energy-filtered TEM (EFTEM) image for Al (Figure 2c) is shown for the final state after 50 h of aging at 750 °C.

The structure of the larger spots in the diffraction pattern in Figure 2a demonstrates a (011) zone axis of a face-centered cubic (FCC) crystal structure arising from the matrix. Less pronounced superstructure diffraction spots can be identified between the large diffraction spots, indicating the presence of a second, ordered cubic phase with an L1_2_-structure. DF imaging in Figure 2b shows that they correspond to the precipitates, as the precipitates have a bright appearance. Furthermore, as shown in Figure 2c by EFTEM, these particles are enriched in Al. 

In general, the investigations reveal that the respective alloy consists of a γ/γ’ microstructure after 50 h of aging at 750 °C, without any other intermetallic phase. Furthermore, this is despite the fact that the CCA under investigation has an increased Al content.

#### 3.1.2. Evolution of γ’ Precipitates as a Function of Tempering Duration

**Figure 3:**
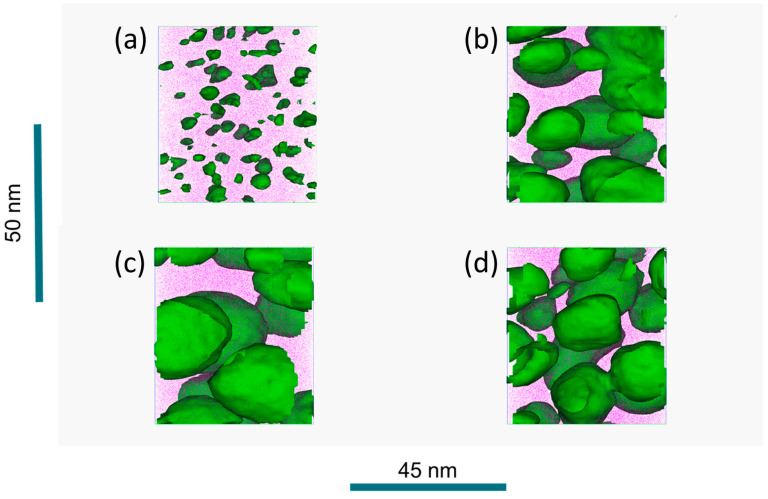
APT reconstruction of a measured volume in WQ state (**a**), 10 h (**b**), 30 h (**c**), and 50 h (**d**) aged state (all after homogenization and annealing). Small (approximately 3 nm sized) Ni and Ti-rich precipitates in the main present matrix phase can already be seen in the WQ state, γ’ precipitates of average size between 11 and 15 nm can be observed in the aged states. Matrix channels with approximately 10–30 nm width can be seen between the precipitates in the aged states.

**Figure 4:**
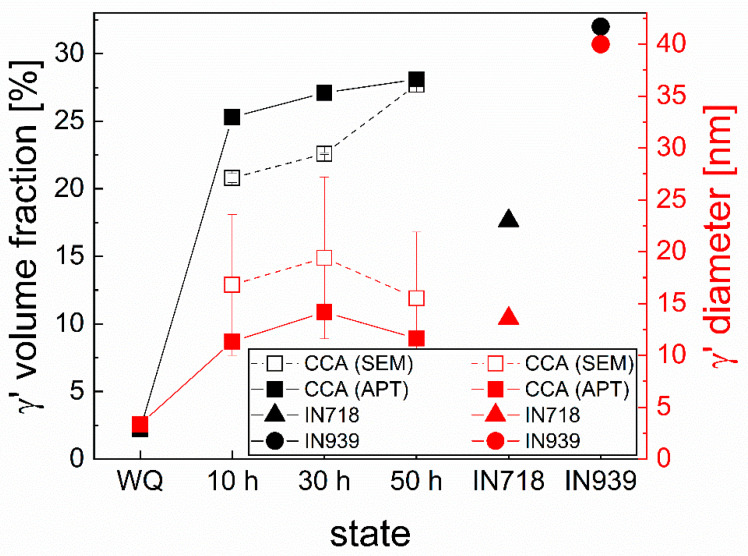
Volume fraction and size of the γ’ precipitates in WQ and aged states (all after homogenization and annealing) from SEM and APT measurements compared to literature data for the Ni-based alloys IN718 and IN939 [23,24,25].

For the detailed microstructural and chemical characterization of the γ’ phase, APT experiments have been carried out in addition to SEM and TEM. A determination of their volume fraction by APT (Figure 3 and Figure 4) shows an increase of the volume fraction from 2 vol.% in the WQ state up to approximately 26 vol.% after 10 h to approximately 28 vol.% after 50 h of aging. Additionally, SEM investigations confirm this trend with much higher statistics, whereby SEM tends to show lower proportions due to limited resolution. The average sizes of the precipitates are determined between 3 nm in the WQ state and 11 to 15 nm for the three aged states via APT, whereby SEM again confirms the trend. 

It should be noted that the APT measurements reveal small (approx. 3 nm average size, with variation between 1 and 5 nm) enrichments of Ni and Ti in the WQ state (Figure 3a), implying a very rapid formation of these γ’ precipitates during quenching. This leads to the assumption that this material can hardly be produced in a totally supersaturated state. For the aged states, average sizes of 11, 14, and 12 nm for 10, 30, and 50 h aging, respectively, with a variation from approximately 7 to 20 nm in each state, are observable in Figure 3b–d. These sizes significantly exceed those of the WQ state. However, an aging time between 10 and 50 h does not lead to a significant increase in their size, as shown by APT and SEM in Figure 4, indicating exceptionally low coarsening kinetics of the precipitates at 750 °C. From the evaluation of the APT measurements, it can also be stated that the volume fraction of γ’ precipitates changes insignificantly during aging between 10 and 50 h, implying a state near the thermodynamic equilibrium already after 10 h of aging. 

The comparison of the size and volume fraction of the CCA’s γ’ particles with the ones from IN718 and IN939 [23,24,25] in Figure 4 shows that the volume fraction of the γ’ phase is higher in the CCA than in IN718. However, it has to be mentioned that in IN718, there is an additional volume fraction of 11 vol.% of the γ’’ phase, which increases the overall precipitate volume fraction to 28.92 vol.%. That is comparable to the 28.1 vol.% precipitate volume fraction in the 50 h aged CCA. The volume fraction of γ’ in the IN939 alloy is 32 vol.%, which is already comparable to the 28.1 vol.% in the CCA. The γ’ size in the IN718, aged at a lower temperature than the CCA, is with 13.5 nm diameter in the same approximate range as in the CCA, whereas the secondary γ’ precipitates in IN939 [24] with approx. 40 nm are found to be substantially larger than the precipitates in the CCA at any considered aging time.

**Figure 5:**
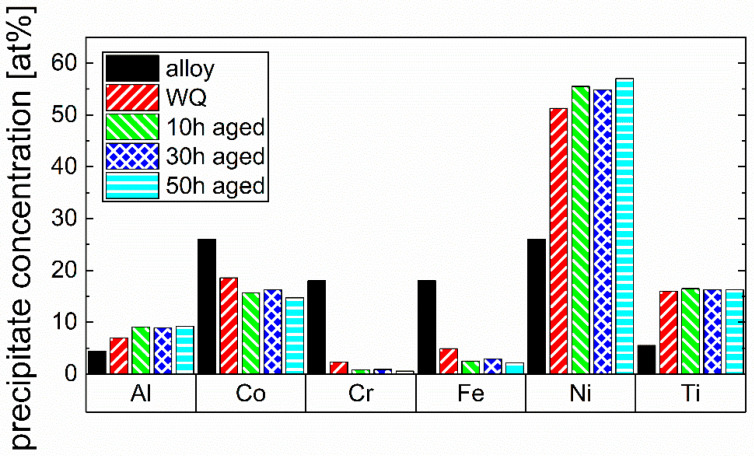
Comparison of the nominal alloy composition (alloy) with the chemical compositions of the precipitate phase in the WQ and the three aged states (all after homogenization and annealing) of the CCA. An accumulation of Al, Ti, and Ni and a depletion of Co, Cr, and Fe can be seen in the precipitate phase with aging.

Aside from size and volume fraction, the evolution of the composition of the γ’ precipitates during aging is also analyzed in detail in this work, as shown in Figure 5. A lower amount of Al and Ni and a higher amount of Co, Cr, and Fe can be observed in the particles of the WQ state compared to the three aged states, even if the Al content is already increased compared to the overall alloy composition in WQ state. The Ti content is rather constant for all states. Despite the difference in the particle’s composition between WQ and aged states, it has to be mentioned that also in the WQ state, the ratio of Al + Ti to Ni + Co is approximately 1 to 3, indicating the stoichiometric formation of a (Ni,Co)_3_(Ti,Al) γ’ phase with minor additions of Fe and Cr. It should be noted that precipitates in a very early stage and small size can deviate in their chemical composition from final precipitates. The γ’ phase has a very similar composition in all three aged states. This further underlines the assumption that the majority of the thermodynamically stable precipitates are already formed after 10 h of aging.

**Figure 6:**
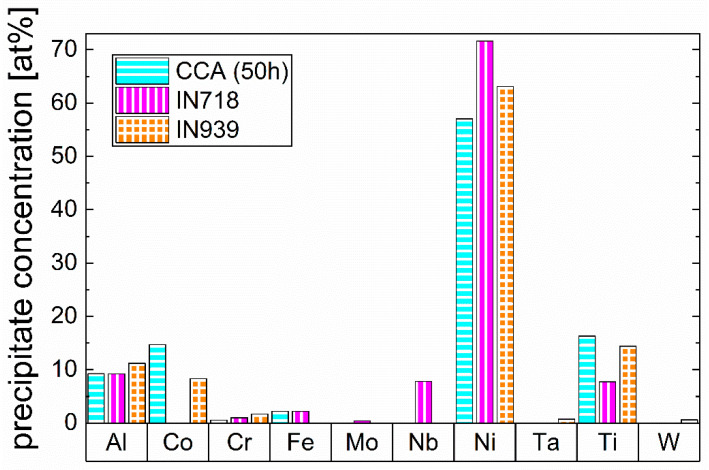
Comparison of the chemical composition of the precipitate phase of the CCA after 50 h of aging with the γ’-composition of Ni-based IN718 [20] and IN939 [21] alloys from the literature.

A comparison of the chemical composition of the γ’ precipitates in the CCA after 50 h of aging to the Ni-based IN718 [20] and IN939 [21] from the literature is observable in Figure 6. This comparison is performed to address the significant chemical differences in this phase between the CCA and the conventionally used alloys, which can influence the properties of the phase. It shows that in the CCA and IN939, Ni sites are also occupied by a significant amount of Co, whereas the investigated CCA shows the highest amount of Co. The sum of Ni and Co amounts to approximately 75 at.% in each alloy, while the sum of Al and Ti for the CCA and IN939, and additionally, Nb for IN718 is approximately 25 at.%. Generally, it can be stated that the stoichiometry of the γ’ phase is (Ni,Co)_3_(Ti,Al) in the CCA, with approximately 15 at.% Co substituting Ni, which is a much larger amount than in IN939 and IN718 (IN718 has no Co at all). Another aspect to mention is that in the CCA, the ratio of Al and Ti in the precipitates is shifted more towards Ti compared to IN939 and IN718 (Ti and Al concentration in the CCA’s precipitates are 16.3 at.% and 9.2 at.%, respectively). Furthermore, it has to be mentioned that Fe and Cr are also found in the CCA’s precipitates, but in a very small amount, with 2.2 at.% and 0.6 at.%, respectively. In IN718, Nb is additionally enriching in the γ’ phase, occupying Al sites [37], which leads to a Ni_3_(Al,Nb,Ti) γ’ phase.

#### 3.1.3. Evolution of the Matrix as a Function of Tempering Duration

From SEM observations of the WQ state in Figure 1b, a supersaturated state, with solely matrix phase present, has been assumed. In contrast to this SEM analysis, it must be emphasized that it is not possible to produce states in the supersaturated solid solution state with a pure matrix phase when considering APT measurements, even with a rapid water-quench, as shown in Section 3.1.2 (Figure 3a). In the aged CCA, the matrix phase is present in channels between the precipitates with channel widths of approximately the same sizes as the diameter of the surrounding precipitates in the bulk of the material, observable from SEM, TEM, and APT (Figure 1c–e, Figure 2c and Figure 3b–d). 

**Figure 7:**
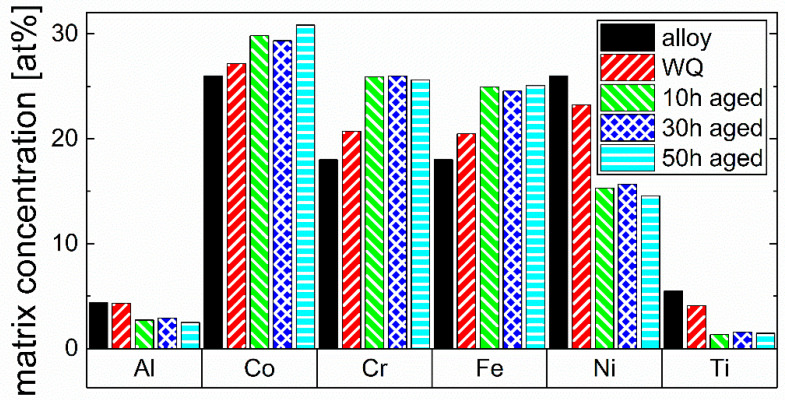
Comparison of the nominal alloy composition (alloy) with the chemical compositions of the matrix phase in the WQ and the three aged states (all after homogenization and annealing) of the CCA. A depletion of Al, Ti, and Ni and an accumulation of Co, Cr, and Fe can be seen in the matrix phase with aging.

In order to investigate the evolution of the matrix composition during aging, the APT measurements have been used again. By excluding the precipitates and diffusion zones, identified as 26 at.% Ni isoconcentration surface, the matrix composition is determined for the respective alloy states. The results of the WQ and the three aged states are compared to each other and to the overall alloy composition in Figure 7.

It reveals a depletion of Al (−1.9 at.%), Ti (−4.0 at.%), and Ni (−11.5 at.%) and an accumulation of Co (+4.8 at.%), Cr (+7.6 at.%), and Fe (+7.0 at.%) in the matrix phase of the 50 h aged alloy as compared to the overall alloy composition. All three aged states show a nearly constant content of all elements without significant changes, indicating a state close to the thermodynamic equilibrium at 750 °C. A different effect can be seen in the WQ state, indicating still a partly supersaturated condition in Al, Ti, and Ni. The Al concentration in the matrix of the WQ state is nearly as high as the concentration in the overall composition, whereas all other elements already change significantly but still do not achieve the composition of the aged states.

**Figure 8:**
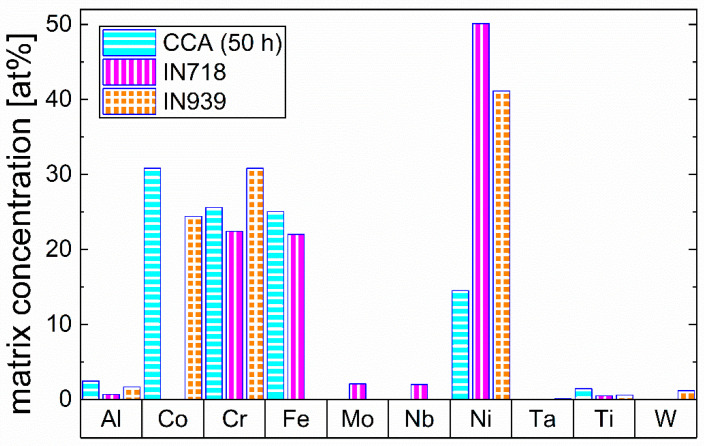
Comparison of the chemical composition of the matrix phase of the CCA after 50 h of aging with the matrix composition of Ni-based IN718 [20] and IN939 [21] alloys from the literature. Significant differences between the matrix compositions can be seen.

Furthermore, the matrix composition of the respective alloy in the 50 h aged state is compared to the matrix compositions of IN718 [20] and IN939 [21] from the literature in Figure 8 to address the significant chemical differences of the matrix between the CCA and already conventionally used alloys. This is important to note, as these differences can affect the properties of the phase in the respective alloy.

The comparison shows significant differences in the matrix composition between the alloys. Whereas the matrix of the respective CCA consists mainly of Co, Cr, Fe, and Ni (30–14 at.%), the Ni-based superalloys exhibit Ni concentrations of 40–50 at.% in the matrix. In the CCA, Co is the main element in the matrix (~32 at.%), and even Cr and Fe have higher concentrations than Ni. Regarding Al and Ti, both elements have higher concentrations in the CCA’s matrix compared to that of both Ni-based superalloys. 

In order to evaluate whether a truly increased entropy is present in the matrix of the respective CCA, this work calculates the APT-informed configurational entropy by using the determined matrix compositions, shown in Figure 8, according to the following Equation (1) [1]:

(1) $${\Delta S}_{conf}=-R\sum_{i=1}^{n}X_{i}\ln X_{i}$$

where *n* is the number of elements, ${\Delta S}_{conf}$ is the ideal configurational entropy per mole, *R* is the ideal gas constant, and *X_i_* is the mole fraction of the *i*^th^ component (i.e., element). Despite the precipitation of particles, the calculations confirm an increased configurational entropy in the matrix of the investigated CCA, as for ${\Delta S}_{conf}$, values of 1.49 *R*, 1.24 *R*, and 1.23 *R* for CCA, IN718, and IN939 are found, respectively. From these results, it can be stated that the true configurational entropy of the CCA’s matrix is significantly higher than in IN939 or IN718 and is directly located at the limit of 1.5 *R* between medium and high entropy.

### 3.2. Prediction of Matrix and Particle Properties

#### 3.2.1. Prediction of Thermodynamic Properties of the Matrix Phase

**Figure 9:**
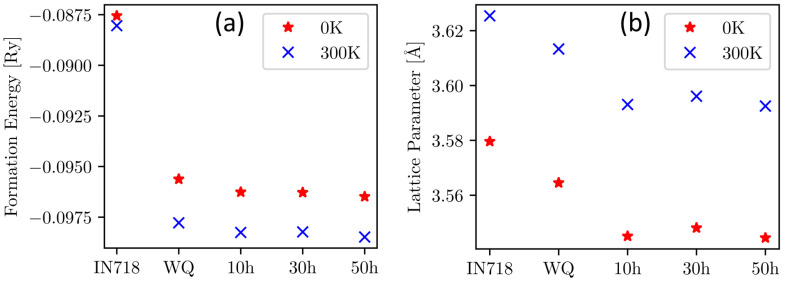
Comparison of calculated material properties for the matrix composition of IN718 from literature ([20]) and the matrix phase of the actual CCA right after water quenching (WQ) and after 10 h, 30 h, and 50 h of aging at 0 K and 300 K. (**a**) Shows the formation free energies, (**b**) the equilibrium lattice constants.

Based on the chemical composition determined by APT measurements, the evolution of the basic properties of the matrix phase in the CCA after WQ and after 10 h, 30 h, and 50 h of aging has been calculated using ab initio methods. Figure 9 shows the calculated lattice parameter and free energy of formation in the considered states and compares them with calculations performed for the composition of the matrix phase of the Ni-based superalloy IN718 found in [20] after 8 h of aging at 718 °C.

The formation energies of the FCC phase in Figure 9a exhibit lower values for all states of the CCA compared to IN718, implying increased thermodynamic stability. While the free energy of formation of the FCC phase in the CCA noticeably decreases from WQ to 10 h of aging, it stays nearly constant with further aging, agreeing with only minor changes in the chemical composition of the matrix. This effect is even more pronounced when looking at the calculated lattice parameters of the matrix phase in Figure 9b. Its value significantly decreases from 3.613 Å to 3.593 Å for 300 K during the first aging step from WQ to 10 h and is again staying rather constant with further aging. The comparison to IN718 shows a higher value for the lattice constant of FCC in IN718 alloy compared to all states of the CCA. The influence of temperature on the formation energy can be seen in Figure 9a, where all values at 300 K are lower than the corresponding values at 0 K, showing stabilization of FCC with increasing temperature. Conversely, the lattice parameter, shown in Figure 9b, increases from 0 K to 300 K by approximately 0.04 Å due to the thermal expansion of solid matter [38]. It should be noted that the trends for the comparison to IN718 are qualitatively the same for both considered temperatures.

#### 3.2.2. Prediction of Elastic and Plastic Properties in the Matrix Phase

**Figure 10:**
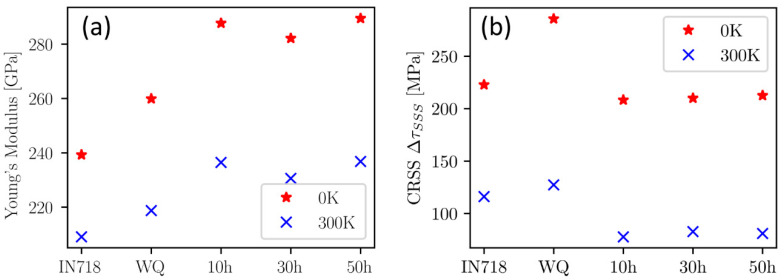
Comparison of calculated materials properties for the matrix composition of IN718 from literature ([20]) and the matrix phase of the actual CCA right after water quenching (WQ) and after 10 h, 30 h, and 50 h of aging at 0 K and 300 K. (**a**) Shows Young’s modulus and (**b**) the critical resolved shear stress (CRSS) [33].

Figure 10 shows the calculated Young’s modulus and critical resolved shear stress (CRSS) of the matrix phase for the different heat treatment states, where the results are also compared to IN718. Young’s modulus is obtained by DFT calculations as described in [35], and the CRSS is derived according to the contribution of the solute-dislocation interaction according to the Varvenne-Curtin model [33], based on the results of DFT calculations of the elastic moduli and misfit volumes [32]. 

Comparison to IN718 alloy from [20] after 8 h of aging at 718 °C in Figure 10 shows significant differences between the alloys. Apart from having smaller lattice constants and being more thermodynamically stable (see Section 3.2.1), the matrix phase of the CCA also has a higher Young’s modulus in all states compared to the matrix phase of the Ni-based IN718 (Figure 10a). The Young’s modulus of the CCA matrix phase increases from the WQ to the 10 h aged state, whereas it stays constant with a longer aging time as the chemistry changes only insignificantly. For the CRSS in Figure 10b, the values for the WQ state are the highest and exceed those for the IN718. With the decrease of CRSS from WQ to the aged states, the CCA shows approximately the same values as IN718 for 0 K and somewhat lower values for 300 K.

#### 3.2.3. Estimation of Yield Strength Contributions 

Besides the CRSS of the FCC matrix phase, also other factors significantly contribute to the yield strength of the investigated CCAs. The CRSS of the pure matrix phase can be mainly assigned to the HEA-type SSS (Δσ*_SSS_*). Additionally, particle strengthening (Δσ*_OS_*) and grain boundary strengthening (Δσ*_HP_*) contribute to the CCA’s yield strength; all contributions can be added up to the final alloy yield strength $\sigma_{y}$. It has to be mentioned that cold-work strengthening based on dislocations can be neglected as all investigated materials have been tested and characterized in the cast and heat-treated state without deformation. Regarding particle strengthening, ordering strengthening (OS) is expected to be the dominant mechanism at room temperature due to the ordered structure, size, and coherency of the precipitates. The atomic ordering contribution has already been determined as decisive for similar alloys and γ’ sizes in the same range as in the present work and up to approximately 25 nm [18,39]. This assumption allows the implementation of Equation (2) to estimate the strength contribution from ordered precipitates, as shown in different works [18,39,40,41,42]. Here, especially the antiphase boundary (APB) energy is a decisive factor.

(2) $${\Delta \sigma }_{OS}=M\times 0.81\frac{\gamma_{APB}}{2b}{\left(\frac{3\pi f}{8}\right)}^{\frac{1}{2}}$$

*M* is the Taylor factor with a value of 3.06 [39,43], *γ_APB_* the APB energy of the γ’ precipitates (calculated to 0.163 J/m^2^ from the ratio of Al and Ti in the precipitates with a model from [44]), *b* the burgers vector, which is $b=a*\frac{\sqrt{2}}{2}$ for FCC structures [45], and *f* is the volume fraction of the precipitates taken from the APT measurements in this work. The lattice parameter a is taken as 0.3572 nm, obtained from XRD measurements (not shown here). For the calculation of Δσ*_HP_*, the conventional Hall-Petch relationship can be used [46,47]:

(3) $${\Delta \sigma }_{HP}=\frac{k_{y}}{\sqrt{d}}$$

where *k_y_* is the strengthening coefficient (a value of 750 MPa×μm^1/2^ is chosen, usual for Ni-based alloys [48]) and d is the diameter of the grains. As the grains in this alloy have dimension sizes in millimeters (see Section 3.1.1), *d* is set to 1000 μm constantly for all states, which leads to a constant contribution of 24 MPa. For ${\Delta \sigma }_{SSS}$, the calculated ${\Delta \tau }_{SSS}$ values from Section 3.2.2 have to be multiplied by the Taylor factor M to get the corresponding yield strength contribution for an equiaxed FCC polycrystal [49]. All the considered contributions and the resulting $\sigma_{y}$ are given for all investigated states in Table 2. Despite the fact that such models are usually used for the prediction of the tensile yield strength, they are compared to the experimentally determined compression yield strength of the 50 h aged state here in order to validate the used parameters. It must be stated that comparability between tensile and compressive yield strength is assumed here, as seen in certain Ni-based alloys (e.g., CMSX-4 or TMS-75 [50]).

From Table 2, it is observable that the predicted yield strength comes close to the experimentally determined 819 MPa, suggesting good applicability of the used models and parameters. As already anticipated from microstructural investigations, the total yield strength of the aged states increases moderately with a difference of 33 MPa between 10 h and 50 h aging at 750 °C. Furthermore, calculations reveal a significantly lower yield strength of 542 MPa for the WQ state, which is 190 MPa lower than for the 50 h aged state. 

## 4. Discussion

This work reveals the evolution of the promising Al_4.4_Co_26_Cr_19_Fe_18_Ni_27_Ti_5.6_ CCA regarding the exact constitution of phases during the favored aging at 750 °C and correlates the results with elastic and plastic properties to understand not only strengthening mechanisms in CCAs but also the alloy’s behavior and chemical constitution during aging.

### 4.1. Alloy Constitution

By a combination of TEM and SEM investigations, this work directly demonstrates that the investigated Al_4.4_Co_26_Cr_19_Fe_18_Ni_27_Ti_5.6_ CCA is constituted by only two phases. These are the FCC matrix and the L1_2_ structured γ’ precipitates, evidenced by an FCC pattern in the (011) zone axis and superstructure peaks in TEM SAD, which is well known from γ/γ’ Ni-based superalloys [51]. Aging for 50 h at 750 °C does not lead to the formation of other TCP phases, such as the sigma phase. Results generally agree with findings revealed by Chang et al. [17] at an Al_3.31_Co_27.27_Cr_18.18_Fe_18.18_Ni_27.27_Ti_5.78_ alloy, suggesting that additional Al combined with a somewhat lower amount of the matrix elements, such as Co, Cr, Fe, and Ni, does not have any effect on CCA’s phase constitution and the formation of TCP phases (e.g., B2 phases or others).

### 4.2. Matrix—Properties and Evolution during Aging

From SEM, TEM, and APT measurements, described in Section 3.1, it is observable that the channel widths of the matrix in the three aged states appear approximately in the same sizes as the diameter of the surrounding precipitates. These channel widths are significantly larger in the WQ state, with approximately 5–25 nm in size. Despite the formation of fine Ni/Ti-rich particles already in the WQ state, the observed difference in the matrix composition between the WQ and the aged states in Figure 7 implies still a supersaturated state, as the amount of measured Al is nearly similar to the melt composition and the Ti content is higher than in the aged states. However, a typical solution annealed state, with all elements dissolved within the matrix, cannot be achieved even with water quenching, causing a significant chemical change from the melt composition. The fact of nearly the same Al concentrations and, at the same time, significantly depleted Ti concentrations between as-quenched matrix and previous melt concentrations suggest that Ti may play an even more important role in the early stages of precipitation of γ’ particles during quenching than Al. After 10 h of aging, the equilibrium seems to be almost reached, and the composition of the matrix does not change substantially with further aging until 50 h. 

The comparison of the matrix compositions between CCA and Ni-based superalloys IN718 [20] and IN939 [21] in Figure 8 reveals substantial differences between the alloys. In the CCA’s matrix phase, Co is the dominant element, whereas Ni still shows the highest concentration in the others. Furthermore, Al and Ti contents also increased compared to the Ni-based superalloys, agreeing with the fact that the approximately 0.25 R higher configurational entropy in the CCA leads to an approximately 2.13 kJ/mol, (i.e., 0.0016 Ry for comparison with Figure 9a) lower free energy of formation at 750 °C. This implies a lower susceptibility for the formation of intermetallic phases (e.g., γ’ (Ni,Co)_3_(Ti,Al)) [52] and may allow increased amounts of Al and Ti to remain in the matrix. In this assumption, only the entropy is taken into account (where ΔG = ΔH − TΔS), but this fact is also supported by computational results in Figure 9a, showing lower formation energy of the CCA’s matrix in all states when compared to IN718. Consequently, according to the results of this work, an increase of Ni at the expense of Co would be interesting, as it would lead to a nearly equiatomic matrix, further increasing the configurational entropy in the matrix and possibly further lowering its free energy of formation. This could lead to a matrix that is even less prone to the precipitation of detrimental intermetallic phases [8]. In contrast, it can promote the precipitation of the Ni-rich γ’ phase, leading to higher volume fractions of this phase, thus, improving precipitation strengthening. It would also lead to a more pronounced contribution to the so-called high entropy core effects [53,54] and improved stability of the matrix phase, according to the PHACOMP approach [55], as Co has a higher electron vacancy number compared to Ni [56]. 

Computational results show that annealing causes a decrease in lattice parameter (Figure 9b) and CRSS (Figure 10b) but a simultaneous increase in Young’s modulus (Figure 10a), especially between WQ and 10 h of aging. This change in properties is caused by the depletion of Ti and Al in the matrix [57,58], forming γ’ precipitates. Compared to IN718, the respective CCA shows lower values for the lattice parameter while simultaneously exhibiting an increased Young’s modulus and comparable CRSS. This combination makes the respective CCA even more promising, as a smaller lattice parameter (leading to a smaller atomic volume) may indicate lower stacking fault energy (SFE) values [59]. Both increased Young’s modulus and low SFE are beneficial for high-temperature properties such as creep resistance of the material [9,60]. The calculated CRSS values of the CCA after 10 h of aging are comparable to the one of IN718, indicating a similar ${\Delta \sigma }_{SSS}$ contribution to the total yield strength in both alloys. However, the studied CCA does not contain Mo or W in the matrix, which are highly active solid-solution-strengthening elements [61]. 

### 4.3. γ’ Precipitates—Properties and Evolution

Ni/Ti-rich particles with approximately 3 nm diameter and a volume fraction of 2 vol.% are observable in the WQ state from APT measurements shown in Figure 3a, indicating a rapid formation during quenching. Such small precipitates are also found in the solution-annealed state of the Al_3.31_Co_27.27_Cr_18.18_Fe_18.18_Ni_27.27_Ti_5.78_ alloy [18]. This rapid formation may be indicative of short-range ordering (SRO) effects present, particularly between Ni and Ti, as these small particles already have high concentrations of these elements. It can be assumed that SRO may serve as a precursor for long-range ordered precipitates (LRO) formed during quenching and subsequent tempering [62]. According to Owen et al. [63], SRO can be distinguished between statistical and microdomain types of SRO. The authors assume that with cooling from the solution annealing temperature, at first, a statistical SRO and then micro-domain SRO is present as a precursor phase, leading to the immediate formation of long-range ordered (Ni,Co)_3_(Ti,Al) precipitates. Additionally, compared to the aged states, the precipitate’s composition of the WQ state is shifted somewhat towards Co and Ti, as Co still has an increased amount and Al is not as strong enriching in the precipitates as Ti. It is well-known that Al usually plays a key role in forming γ’ precipitates, but Ti increases their thermal stability in Co-Ni-based alloys by increasing the solvus temperature [64], leading to a possible accelerating effect of Ti on their formation. It must be mentioned that for all states, the ratio of Al + Ti to Ni + Co is approximately 1–3. This is in accordance with the nominal composition of the (Ni,Co)_3_(Al,Ti) γ’ phase, known from Ni-Co-based superalloys, containing Al and Ti [65,66,67], evidencing that even the smallest particles in the WQ state seem to already be L1_2_ γ’ precipitates. These mechanisms might also be transferable to Ni-based superalloys, as a rapid formation of precipitates is already reported in, for example, ATI Allvac 718Plus [68] or other CCAs [69]. However, the true evidence of the formation mechanism and the role of SRO effects before precipitation of γ’ precipitates should be the scope of future work. The shift of the γ’ precipitate composition towards Co and Ti in the WQ state evidences the stability of the continuous γ’ phase-field between Ni_3_Al and Co_3_Ti [70] even in the actual, complex high-entropic alloy composition. According to [67,71,72], the incorporation of Co and Ti can improve the high-temperature strength in the Ni_3_Al phase, as beyond 730 °C Co_3_Ti exhibits higher strength than Ni_3_Al. Compared to the Ni-based superalloys, the γ’ phase in the respective CCA has higher amounts of Co and Ti, suggesting its high potential for better mechanical behavior at higher temperatures.

According to Figure 4, the 3 nm precipitates grow in bulk during aging to a size of about 11–15 nm and a volume fraction of 25–28 vol.% and show a spherical shape. Compared to the Al_3.31_Co_27.27_Cr_18.18_Fe_18.18_Ni_27.27_Ti_5.78_ alloy with a lower amount of Al from [17], showing 25 nm sized precipitates and a volume fraction of approximately 34 vol.% for the γ’ phase after 50 h of aging at 750 °C, the actual alloy has smaller sizes and a somewhat lower volume fraction of the precipitates in the aged state. The evolution in Figure 4 shows that the formation of the particles in the previously mentioned size and volume fraction occurs especially during the first 10 h and remains approximately stable within the next 40 h of aging, leading to a final precipitate size similar to conventionally processed IN718 [23]. This behavior is known from Ni-based superalloys, such as IN718, showing an increase in the size of their γ’ precipitates, especially in the first hours of aging [73]. 

Conversely, a comparison to the literature and IN718, aged in the same conditions as the CCA (750 °C and holding time of 50 h), reveals a somewhat larger size of 30 nm of the γ’ precipitates and constant growth of the particles between 30 and 200 h of aging [73], indicating possible lower coarsening kinetics and increased stability of the γ’ precipitates in the investigated CCA compared to IN718. This trend remains valid when compared to other Ni-based superalloys, such as IN939 alloy [24], as the secondary γ’ precipitates in IN939 (i.e., those that precipitated in the last aging step at a somewhat higher temperature of 800 °C for 16 h) with approximately 40 nm diameter, are substantially larger than the precipitates in the CCA at any considered aging time. This implies that the difference is not only due to the aging temperature and time but also faster growth of the precipitates in the Ni-based alloy. The high stability and resistance against particle coarsening agree with the Al_3.31_Co_27.27_Cr_18.18_Fe_18.18_Ni_27.27_Ti_5.78_ alloy, where Ming et al. [19] also reported their high stability. High stability of the precipitates against coarsening can be caused by a low lattice misfit between the phases, as the driving force for coarsening is related to the interface energy, as well as a low diffusion coefficient in the matrix [9,74,75], which seems to be optimized in the respective CCA compared to the benchmark of Ni-based superalloys, such as IN718 or IN939. However, aside from the particles in bulk, SEM investigations in Figure 1f show coarsened and elongated precipitates with lengths of up to 650 nm and widths of up to 100 nm in the proximity of grain boundaries. Such behavior of precipitates has also been found in the Al_3.31_Co_27.27_Cr_18.18_Fe_18.18_Ni_27.27_Ti_5.78_ alloy [36]. According to [36], this is because of enhanced growth of the precipitates and accelerated diffusion in this region, causing an elongated shape of these precipitates because of grain growth and related grain boundary movement during aging. These regions appear to be weak spots in the material, leading to the conclusion that the ambition to prevent this behavior would be beneficial for the material’s mechanical properties. Thus, it is suggested to decrease the mobility of the grain boundaries as well as the diffusivity of elements at the grain boundaries by Zener or solute drag effects [76,77,78]. This can be realized by carbides or other intermetallic particles alongside grain boundary active elements [36,79,80].

### 4.4. Yield Strength Contributions 

Yield strength contributions were estimated in this work and compared to the experimental compression yield strength of the CCA aged at 750 °C for 50 h, shown in Table 2. In general, the yield strength calculation with 732 MPa is in the range of the experimental results, indicating an appropriate choice of parameters in these calculations. However, a difference of 87 MPa between the calculated and experimental results is identified, which may arise due to the difference between compression and tensile yield strength, which is more often predicted by these models. Additional mechanisms for particle strengthening, such as the coherency effect, modulus effect, stacking fault effect, or interfacial effect [81], are assumed to be negligible in this work, which, however, can increase the precision of the yield strength prediction. Parameters, such as shear moduli of matrix and precipitates, the lattice parameter of the precipitate, energy of the precipitate-matrix interface, and others, needed for prediction of these effects in this alloy, are thus planned to be determined in future work to increase the model’s precision. Furthermore, in the present work, the APB energy was calculated to 0.163 J/m^2^ from the ratio of Al and Ti in the precipitates by a model for a Fe-Ni-Cr alloy [44], which might not precisely fit CCAs. In this model, Co is not considered for increasing the APB energy, as suggested in work dealing with IN939 [82]. Moreover, even for the APB energy of the pure Ni_3_Al phase, there is a significant variation in the literature [83], leading to an uncertainty of the exact value and justifying slight changes in the original value. However, when neglecting mechanisms other than the ordering strengthening, actual results would suggest an increased value of 0.194 J/m^2^ for the APB energy of the (Ni,Co)_3_(Al,Ti) γ’ phase, resulting in a total yield strength of 820 MPa of the CCA aged at 750 °C for 50 h. This value is reasonable for such kinds of alloys since Dasari et al. [18] used an APB energy of 0.19 J/m^2^ for estimating the yield strength in the Al_3.31_Co_27.27_Cr_18.18_Fe_18.18_Ni_27.27_Ti_5.78_ alloy and calculated a yield strength of 848 MPa for the aged state, which corresponds well with their experimental value and also with the results of the present work. However, the exact determination of APB energies in precipitation-strengthened CCAs should be the scope of future work.

In order to exclude the possibility that the Orowan mechanism might be the decisive particle-strengthening mechanism for the intragranular precipitates, an estimation of the Orowan stress can be calculated. In [19], the following equation is used for the calculation of the Orowan stress ${\Delta \sigma }_{Or}$:

(4) $${\Delta \sigma }_{Or}=\frac{Gb}{2\pi l}ln\left(\frac{l}{r_{0}}\right)$$

where *G* is the shear modulus, *b* is the Burgers vector, *l* is the average edge-to-edge inter-precipitate distance, and *r*_0_ is the dislocation core radius (*r*_0_ ≈ *b*). The average edge-to-edge inter-precipitate distance for the aged states in the present work can be approximated as the matrix channel width, which is approximately equal in size to the precipitate’s diameter, as stated in Section 3.1.3. By using Equation (4) with an edge-to-edge inter-precipitate distance of 12 nm, a G of 78.5 GPa [39], the same b as in Section 3.2.3, and an *r*_0_ equal to *b*, ${\Delta \sigma }_{Or}$ yields a value of approximately 1015 MPa. This is a much higher value than the values for the ${\Delta \sigma }_{OS}$ in any state, observable in Table 2. Therefore, it can be concluded that it is much easier for the dislocations to cut the precipitates than to bypass them via the Orowan mechanism, confirming the acting mechanism as cutting as proposed in Section 3.2.3.

It should also be noted that for the calculation of the precipitation strengthening, the discontinuous precipitates in the proximity of grain boundaries, observable in Figure 1f, were not taken into account. This is due to the assumption that with the large grain size in the respective alloy, the overall volume with large discontinuous precipitates is negligibly small compared to the overall intragranular volume, where small precipitates are present.

Consequently, it is assumed in the calculations that the main contributions to yield strength are ${\Delta \sigma }_{OS}$ from the intragranular particles and ${\Delta \sigma }_{SSS}$ from the matrix, given in Table 2. CRSS calculations based on ab initio calculations of the matrix in this work and their derivation to ${\Delta \sigma }_{SSS}$ additionally suggest a higher solid solution strengthening than calculated in the Al_3.31_Co_27.27_Cr_18.18_Fe_18.18_Ni_27.27_Ti_5.78_ alloy by Dasari et al. [18], where they used the Fleischer model for the contribution of solid solution strengthening. However, they used an intrinsic strength from the CoCrFeNi alloy as a starting point and calculated the effect of Al and Ti addition to this alloy. In the CRSS calculations in the present work, such an intrinsic strength would be included already. From the WQ state to the 50 h aged state, a clear decrease of ${\Delta \sigma }_{SSS}$ from 390 MPa to 248 MPa can be seen for the matrix phase. This is primarily due to the depletion of the solid-solution-strengthening elements Al and Ti, as they are incorporated into the precipitates during aging. However, the calculated yield strength of the alloy increases with aging from 542 MPa to 732 MPa due to the precipitation-strengthening effect. Hence, the strength provided by the arising precipitates obviously overcompensates the strength loss from decreasing solid solution strengthening in the matrix during aging.

## 5. Conclusions

In this work, the aging behavior of a promising CCA for high-temperature application from WQ state to a maximum of 50 h aging at 750 °C is characterized by SEM, TEM, and APT measurements. The shape, size, and composition of the matrix and precipitate phases are determined and compared to Ni-based superalloys from the literature. Properties of individual phases have been studied using ab-initio-based model calculations supported by complementary experimental investigations.

The following conclusions can be drawn from the results:

- After 10 h of aging at 750 °C, the majority of the thermodynamically stable γ’ ((Ni,Co)_3_(Al,Ti)) precipitates are formed. A comparison of the precipitate size after 50 h of aging with state-of-the-art Ni-based alloys from the literature suggests a comparatively low coarsening of the particles.
- Ni- and Ti-rich precipitates are observed in APT measurements of the WQ state, which already possess the stoichiometric ratio of the (Ni,Co)_3_(Al,Ti) γ’ phase. This indicates that it is nearly impossible to obtain a fully supersaturated and single-phased (ideal HEA) state in the selected CCA. The rapid formation of the precipitates during water quenching implies that ordering effects may have an active role during phase formation.
- Computational results for the matrix phase predict a promising combination of the low formation energy (i.e., high phase stability), lattice misfit, higher Young’s modulus, and similar CRSS compared to the Ni-based IN718.
- Model calculations show the dominance of the precipitation strengthening mechanism in determining the room temperature yield strength of the aged CCA, which gradually increases with the aging time at 750 °C, and the dominance of the solid solution strengthening mechanism in the WQ state.

## Figures and Tables

**Table 1:** Chemical alloy composition of the investigated CCA and the Ni-based alloys IN718 from [20] and IN939 from [21] in at.-%.

	Ref.	Ni	Co	Cr	Fe	Al	Ti	Nb	Mo	Others
CCA (at.-%)	This work and [22]	26.71	26.34	18.64	17.97	4.39	5.55	-	-	0.40 Rest
IN718 (at.-%)	[20]	53.48	-	19.87	19.29	1.08	1.18	3.19	1.80	0.11 C
IN939 (at.-%)	[21]	46.47	18.23	24.47	-	3.98	4.37	0.61	-	0.62 W
										0.44 Ta
										0.05 B
										0.71 C
										0.06 Zr

**Table 2:** Yield strength contribution from grain size Δσ *_HP_* (calculated with Equation (3)), from solid solution strengthening Δσ *_SSS_* (calculated from CRSS from computational methods), and from particle strengthening ${\Delta \sigma }_{OS}$ (calculated with Equation (2)), as well as the sum of the contributions $\sigma_{y}$, for different states of the CCA compared to the experimentally determined yield strength for the 50 h aged state from the compression test.

	WQ	10 h Aged	30 h Aged	50 h Aged	
	Calculated				Experimental
${\Delta \sigma }_{HP}$ [MPa]	24	24	24	24	-
${\Delta \sigma }_{SSS}$ from CRSS [MPa]	390	238	253	248	
${\Delta \sigma }_{OS}$ [MPa]	128	437	452	460	
$\sigma_{y}$ [MPa]	542	699	729	732	819

## Data Availability

The raw/processed data required to reproduce these findings are available to obtain from the corresponding author.
